# Undiagnosed impacted knife blade from a penetrative orbital injury: A case report

**DOI:** 10.1016/j.ijscr.2018.10.064

**Published:** 2018-11-01

**Authors:** Mohamed El Sayed, Reem Hassan Saad, Ahmed Fereir

**Affiliations:** a Department of Otolaryngology-Head and Neck Surgery, Faculty of Medicine, Benha University, Benha, Egypt; b Department of Cranio-Maxillofacial Surgery, Nasser Institute for Research and Treatment, Cairo, Egypt; c Department of Oral and Maxillofacial Surgery, Faculty of oral and dental medicine, Future University, New Cairo, Cairo, Egypt; d Department of Oral and Maxillofacial Surgery, National Bank Hospital For Integral Care, El Katameya, Cairo, Egypt

**Keywords:** Facial trauma, Stab wounds, Foreign body, Maxillofacial, Orbital injury, Case report

## Abstract

**Introduction:**

Impacted foreign bodies in the complex maxillofacial region is uncommon and their safe removal is a challenge.

**Case presentation:**

The authors report an unusual case of a 41 year old male patient who suffered from violence-related orbital trauma with a knife. The presence of this foreign body was not diagnosed for a period of 20 months. After proper clinical and radiologic examination it was localized and extracted in the theater in a safe controlled manner. Although the tip of the blade was left in situ, the patient had a rapid uneventful recovery and follow up.

**Discussion:**

The path of penetration of the foreign body in presented case didn't follow the predicted patterns of orbital injury described in literature. A dilemma exists as to how aggressive such injuries should be managed. Thus the surgical approach implemented for retrieving the current foreign body was highlighted.

**Conclusion:**

Radiographs are a crucial element for early diagnosis and proper management of foreign body injuries. Rapid postoperative recovery can be achieved with simple safe surgical retrieval maneuvers.

## Highlights

• The mode of entry of the foreign body was unique as well as the force required to impale and break a knife blade in bone.
• The authors present a minimally invasive technique for dealing with this type of injury and highlight its challenges.
• This case report emphasis the need for baseline radiology in cases of traumatology especially those with vague history.
• The patient was examined by multiple physicians who did not detect the foreign body which could raise medico-legal issues.

## 1 Introduction

Violence is a complex, multidimensional phenomenon accounting for an average of 52% of all trauma patients [1]. Knife violence has gained worldwide media attention and is regarded as a weapon of opportunity affiliating serious wounds through different force components. This includes axial, lateral and/or cutting forces with or without a torqueing action. The parameters governing knife stab attacks includes the force required for penetration, the mechanics of stabbing, victim-related factors as well as knife characteristics [2]. The uniqueness of the presented injury is due to the considerable amount of force required to impale and break a knife blade in bone [3]. The presented case report is in line with SCARE guidelines [4].

## 2 Case presentation

This is a case report of a 41 year old male patient who suffered a knife inflicted injury to the face. He was referred to our department 20 months after the incidence complaining of persistent discomfort with downward gaze of the left eye. He reported being agitated and feared a possible sight-threatening condition after the alleged assault. He was transferred to an emergency department where he was examined by an ophthalmologist who reassured him that his globe was intact and sutured the deep cut wounds related to his upper and lower eyelids. He was reassured that the facial edema would subside and the neurosensory deficit he was suffering from would eventually recover. Empirical antibiotics, Analgesia and tetanus prophylaxis were prescribed and the patient was discharged from the emergency department. He was unsatisfied with the resultant scar in his left eyelids and six months later was re-examined by a plastic surgeon that performed blepharoplasty. The persistent complaint of discomfort related to his left eye led him to seek medical advice again before being referred to our department.

**Fig. 1:**
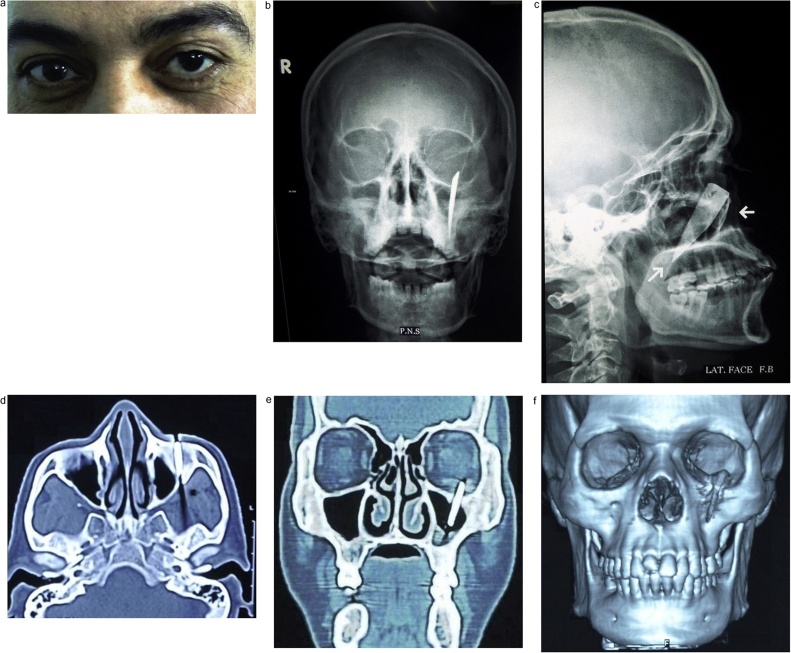
**Showing the preoperative presentation of the patient with the foreign body in situ**. (a) frontal view of the patient with unequal eye level. (b, c) Paranasal sinuses and lateral skull views respectively. (d, e, f) Axial, coronal and 3D computed tomography cuts respectively.

Systematic thorough clinical and radiographic examination was performed. Upon inspection, it was noted that the left globe was displaced superiorly with increased scleral show (Fig. 1a). Bimanual palpation was done and a hard object could be felt behind the orbital rim of the left eye; although it was not visible externally. No evidence of diplopia was noted by ophthalmological examination. Plain radiography (Paranasal sinuses view (P.N.S) and lateral skull view) revealed a radiopaque foreign body (FB) lodged inside the orbital floor and directed downward & posteriorly (Fig. 1b and c). Computed tomography (CT) scan was ordered to precisely localize the foreign body and its relation to the surrounding structures. The foreign body was found penetrating the facial skeleton at the level of the orbital floor and transgressed the maxillary antrum and its tip reaching the pterygoid plates of maxilla (Fig. 1d–f).

**Fig. 2:**
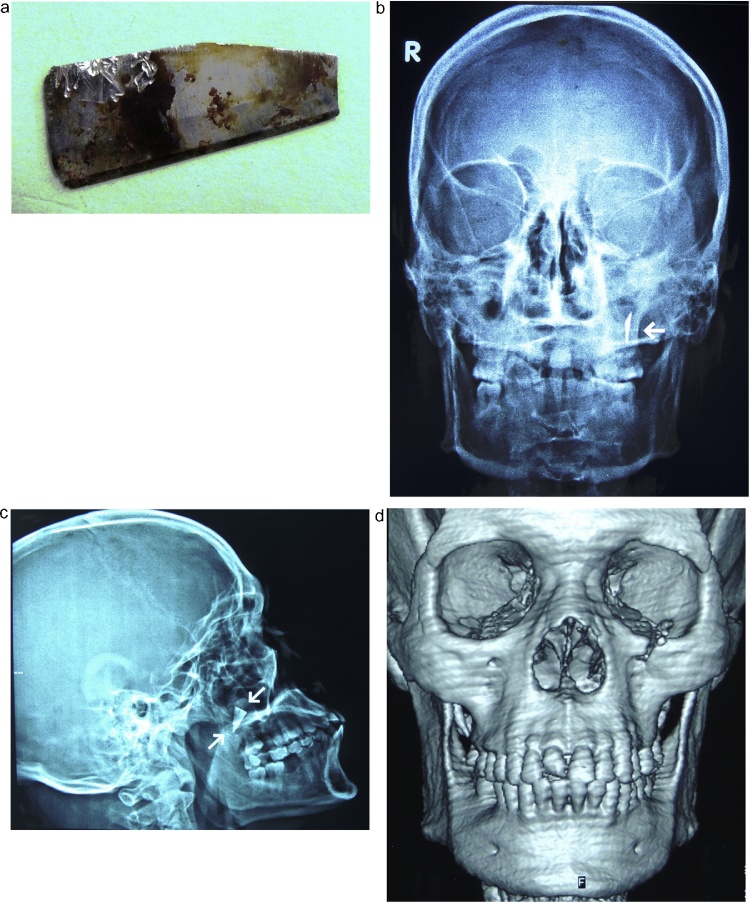
**Showing the immediate postoperative results** (a) the extracted knife blade (the foreign body) (b, c) posteroanterior and lateral skull views respectively showing the retained tip of the foreign body. (d) postoperative 3D computed tomography showing the resultant defect in the inferior orbital rim.

Management of this type of injury has no formally documented guidelines in literature. The decision for surgical removal of this foreign body was made based on the patient's chief complain, general surgical principles and facial esthetics. An extraoral approach under general anesthesia was chosen and a preoperative informed consent was obtained. The orbital floor was exposed through a subciliary incision and the intraorbital portion of the foreign body was found firmly anchored to bone. A vertical osteotomy from the orbital rim to the anterior wall of the maxillary sinus was performed to avoid extraction of the object along its path of insertion. A wire-twister was used to firmly grasp the foreign body which was retrieved without any significant bleeding and was found to be a stainless steel knife blade (Fig. 2a). The orbital floor and rim were carefully evaluated and the need for internal orbital reconstruction was found unnecessary.

At that point it was noted that a portion of the foreign body, considered the blade tip, was not retrieved. A C-arm was used in an attempt to guide its localization and removal but provided insufficient information. The risks of proceeding with a blind surgical approach in the complex maxillofacial region outweighed the benefits of removing the retained foreign body and the decision to terminate the surgery was made. The possibility of returning at a later stage for a more extensive procedure after obtaining a new patient consent and the required radiographs was considered. The patient was discharged the same day of the operation and was regularly monitored in the first two weeks postoperatively (Fig. 2b–d).

**Fig. 3:**
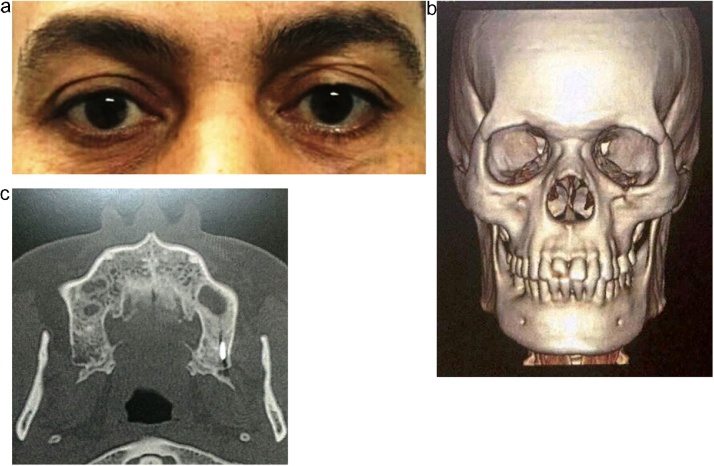
**Showing clinical and radiographic results of the follow up period** (a) Frontal view of the patient at 1 year postoperative with equal eye level. (b) 3D computed tomography at 2 years postoperative showing bone healing at the previous defect in the inferior orbital rim. (c) axial CT cut of the retained foreign body at 2 years of postoperative follow up.

At the first and third months of the follow up period, the patient has made rapid recovery with no signs or symptoms of infection. At 1 year of postoperative follow up, clinical examination showed normal symmetrical eye position and no ectropion (Fig. 3a) as well as improvement in the neurosensory deficit. At 2 years of postoperative follow up, radiographic examination showed bone healing of the previous defect at the orbital rim (Fig. 3b). It also demonstrated that the impacted portion of the foreign body has remained lodged in the exact same position (Fig. 3c). The option of leaving or retrieving the foreign body was presented again to the patient but he ensured us to be extremely satisfied with the outcome and refused to proceed with further surgical procedures.

## 3 Discussion

Slash wounds should be differentiated from stab wounds and clinical examiners should hold a high index of suspicion to stab wounds and a low threshold for requesting plain radiographs. Negative clinical picture despite the presence of a foreign body has been noted by previous investigators [5]. The lack of infection related to the foreign body in our case is in accordance with other studies [6,7]. On the other hand, foreign bodies crossing the maxillary sinus are usually acutely symptomatic unlike our case report [23,24]. Although the portion of the foreign body crossing the maxillary sinus in our case has been retrieved; there is no reported significant correlation between retained metal fragments and infections [20].

The choice between transcutaneous and transconjunctival approaches is controversial [17,18] In the present case report, the surgeons chose a transcutaneous approach to avoid adding any unnecessary risks to the globe and to easily manage any unexpected deviation of the normal orbital anatomy caused by the presence of the foreign body [17,19].

The globe is fairly resilient to trauma due to its tough sclera and relative mobility within the surrounding bed of intraorbital fat [8]. Knowledge of the mechanism and the common patterns of orbital injury should be beneficial. The converging orbital walls usually guide the penetrating object to exit through the superior orbital fissure, inferior orbital fissure or orbital canal towards the nearby vital structures which threatens the patient's life [8,9]. Meanwhile, our case report presents an unusual path of penetration of a low-velocity object that didn't follow the vertical nor horizontal predicted patterns of orbital injury described in literature.

The preferred path of retrieval of impacted foreign bodies for many authors has been along its path of insertion [5,[11], [12], [13]]. In the presented case, the added vertical osteotomy was performed to avoid extracting the blade along its path of insertion (vertically upwards) to protect the globe. Similar challenges regarding the path of retrieval was met and reported in literature [14,15].

A dilemma exists as to how aggressive such injuries should be managed [10] and whether a minimally invasive surgical approach like in our case report or rather a radical technique like orbitotomy [16] would be more beneficial. Although the surgical team is fully capable of retrieving the remaining impacted tip of the foreign body; it is contraindicated to undergo surgical procedures only for removing retained fragments [20] Given its asymptomatic long-term retention, small size, intraosseous location and stainless steel composition, the option of conservative, periodic follow up was presented to the patient and he has repeatedly refused any further surgical maneuvers as his chief complain has already been solved [21,22].

## Conflict of interest

The authors have no conflict of interest of any aspect.

## Sources of funding

The authors had no source of funding and no sponsors to this work.

## Ethical approval

The data presented in the current case report is reviewed and approved by the Ethical Committee at our hospital. The patient signed a "release form" to give the authors the permission needed for publication.

## Consent

A preoperative "Informed consent" was obtained.

The patient also signed a "release form" to give the authors the permission needed for publication.

## Author contribution

**Mohamed El Sayed**

- The consultant physician and operator who performed the surgical removal of the impacted knife blade and the modification from the path of insertion.
- Postoperative Follow up of the patient condition and progress.
- Supervised the development of the work.

**Reem Hassan Saad**

- Postoperative Follow up of the patient progress.
- Acquisition of data.
- Wrote the manuscript and the revised versions.

**AhmedFereir**

.
Postoperative Follow up of the patient progress.
Analysis of data.
Critical revision of the manuscript.

*All authors discussed the results and approved the submitted version of the manuscript*.

## Registration of research studies

This case study was written in accordance to Helsinki guidelines.

## Guarantor

All the authors (Mohamed El Sayed, Reem Hassan Saad, Ahmed Fereir).

## Provenance and peer review

Not commissioned, externally peer reviewed.
